# Inorganic pyrophosphatase in uncultivable hemotrophic mycoplasmas: identification and properties of the enzyme from *Mycoplasma suis*

**DOI:** 10.1186/1471-2180-10-194

**Published:** 2010-07-20

**Authors:** Katharina Hoelzle, Simone Peter, Michele Sidler, Manuela M Kramer, Max M Wittenbrink, Kathrin M Felder, Ludwig E Hoelzle

**Affiliations:** 1 Institute of Veterinary Bacteriology, University Zurich, Winterthurerstr. 270, 8057 Zurich, Switzerland

## Abstract

**Background:**

*Mycoplasma suis *belongs to a group of highly specialized hemotrophic bacteria that attach to the surface of host erythrocytes. Hemotrophic mycoplasmas are uncultivable and the genomes are not sequenced so far. Therefore, there is a need for the clarification of essential metabolic pathways which could be crucial barriers for the establishment of an *in vitro *cultivation system for these veterinary significant bacteria.

Inorganic pyrophosphatases (PPase) are important enzymes that catalyze the hydrolysis of inorganic pyrophosphate PP_i _to inorganic phosphate P_i_. PPases are essential and ubiquitous metal-dependent enzymes providing a thermodynamic pull for many biosynthetic reactions. Here, we describe the identification, recombinant production and characterization of the soluble (s)PPase of *Mycoplasma suis*.

**Results:**

Screening of genomic *M. suis *libraries was used to identify a gene encoding the *M. suis *inorganic pyrophosphatase (sPPase). The *M. suis *sPPase consists of 164 amino acids with a molecular mass of 20 kDa. The highest identity of 63.7% was found to the *M. penetrans *sPPase. The typical 13 active site residues as well as the cation binding signature could be also identified in the *M. suis *sPPase. The activity of the *M. suis *enzyme was strongly dependent on Mg^2+ ^and significantly lower in the presence of Mn^2+ ^and Zn^2+^. Addition of Ca^2+ ^and EDTA inhibited the *M. suis *sPPase activity. These characteristics confirmed the affiliation of the *M. suis *PPase to family I soluble PPases. The highest activity was determined at pH 9.0. In *M. suis *the sPPase builds tetramers of 80 kDa which were detected by convalescent sera from experimentally *M. suis *infected pigs.

**Conclusion:**

The identification and characterization of the sPPase of *M. suis *is an additional step towards the clarification of the metabolism of hemotrophic mycoplasmas and, thus, important for the establishment of an *in vitro *cultivation system. As an antigenic and conserved protein the *M. suis *sPPase could in future be further analyzed as a diagnostic antigen.

## Background

*Mycoplasma suis *belongs to a group of highly specialized uncultivable hemotrophic bacteria within the family *Mycoplasmataceae *that attach to the surface of host erythrocytes [1,2]. In the last few years reports on hemotrophic mycoplasmas in various animal species [1] as well as in humans [3,4] continuously increased. Obviously, hemotrophic mycoplasmas are emerging agents with a zoonotic potential. *M. suis *causes infectious anemia in pigs leading to serious economic loss in the pig industry due to acute anemia as well as chronic persistent infections with increased susceptibility to respiratory and enteric diseases [1,5].

Instead of a clear and long-dated clinical significance of hemotrophic mycoplasmas [6] our knowledge on the physiology and metabolism of hemotrophic mycoplasmas is rather limited. This can primarily led back to their unculturability and the lack of sequence data [6]. Probably, *M. suis *can use glucose as a source of carbon and energy [7,8]. However, detailed energy requirements of *M. suis *are largely unknown and its key enzymes have not been described so far. In previous studies we successfully screened genome libraries to identify *M. suis *proteins which are involved in pathogenetic processes of *M. suis *infections (e.g. adhesion) and the energy metabolism of these rather unexplored pathogens [9,10]. In this paper we identified the soluble inorganic pyrophoshatase (sPPase) of *M. suis *by applying said strategy. Inorganic pyrophosphate (PP_i_) is an important by-product of many biosynthetic processes, and sPPases which hydrolyze PP_i _to inorganic phosphate (P_i_), are essential and ubiquitous metal-dependent enzymes providing a thermodynamic pull for many biosynthetic reactions [11-13]. Soluble PPases belong to two non-homologous families: family I, widespread in all types of organisms [14], and family II, so far confined to a limited number of bacteria and archaea [15,16]. The families differ in many functional properties; for example, Mg^2+ ^is the preferred cofactor for family I sPPases studied, whereas Mn^2+ ^confers maximal activity to family II sPPases [17,18]. Detailed aims of this study were the recombinant production and characterization of the *M. suis *sPPase and the comparison of its properties to those of other bacteria. Characterization of essential enzymes in the metabolism of hemotrophic mycoplasmas are important steps towards the establishment of an *in vitro *cultivation system for this group of hitherto uncultivable hemotrophic bacteria.

## Results

### Identification of the *M. suis *inorganic pyrophosphatase (PPase)

**Figure 1:**
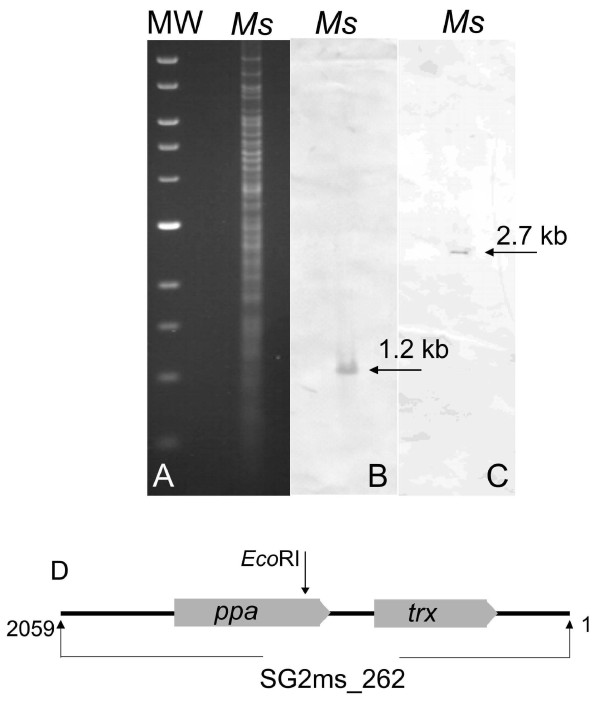
**Southern blot hybridization of *Eco*RI-restricted *M. suis *DNA showing the genomic location of the *ms*262 clone insert on a 1.2- and a 2.7-kb fragment**. (A) agarose gel electrophoresis of *Eco*RI-restricted DNA. (B) the blot probed with the DIG-labeled 950 bp-*Eco*RI fragment of the library clone *ms*262; (C) the blot probed with the DIG-labeled 1050 bp-*Eco*RI fragment of the library clone *ms*262; (M) molecular weight standard; (*Ms*) *M. suis*. The arrows indicate the positions of the hybridized 1.2- and 2.7-kb fragments. (D) schematic map of the ORF localisation on the library clone *ms*262. The grey box arrows indicate the two ORFs: *ppa *(inorganic pyrophosphatase) and *trx *(thioredoxin).

**Figure 2:**
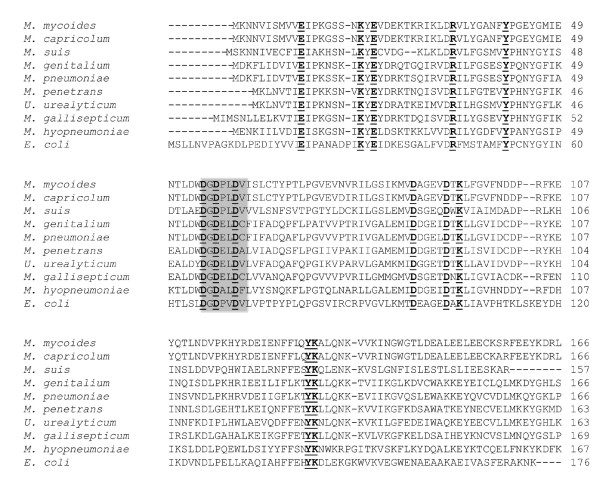
**Alignment of the sPPase sequences of *M. suis*, selected *Mycoplasma *species and *Escherichia coli***. Sequences were aligned using the ClustalW tool http://www.ebi.ac.uk/Tools/clustalw2/. The 13 conserved residues which build the active site (Sivula et al., 1999) are bold-faced and underlined. The residues which are essential for the cation binding are emphasized by a grey box. Accession numbers for the sequences follow: *M. mycoides *ssp *mycoides *SC str. PG1 NC_005364; *M. capricolum *ssp *capricolum *CP000123; *M. suis *FN394679; *M. genitalium *L43967; *M. pneumonia*e U00089; *M. penetrans *NC_004432; *U. urealyticum *serovar 10 NC_011374; *M. gallisepticum *AE015450; *M. hyopneumoniae *NC_007295; *E. coli *NC_010468.

The sPPase of *M. suis *was identified by screening of genomic libraries of *M. suis *using shot gun sequencing. By means of sequence analysis and database alignments of 300 randomly selected library clones we identified library clone *ms*262 containing an *M. suis *insert with highest identity to the gene encoding the *M. penetrans *sPPase. Since prokaryotic sPPases are known to be essential in energy metabolism [11,12] we selected the *ms*262 clone for further studies. To confirm the *M. suis *authenticity of *ms*262 Southern blot analyses of *M. suis *genomic DNA were performed using two *Eco*RI *ms*262 library fragments as probes. The *ms*262 *EcoR*I fragments hybridized with two genomic *M. suis *fragments of 1.2 and 2.7 kb, respectively (Figure 1A). Detailed sequence analysis revealed that the clone *ms*262 contains a 2059-bp insert with an average G+C content of 30.11%. Clone *ms*262 includes two ORFs (Figure 1B): ORF1 showed the highest identity with *U. parvum thioredoxin trx *(significant BLAST score of 1.3 × 10^-7^, overall sequence identity 44.5%). ORF2 with a length of 495 bp encodes a 164-aa protein with a calculated molecular mass of 18.6 kDa and an isoelectric point of 4.72. The ORF2 matched best with *M. penetrans ppa *(63.7% identity). The overall degrees of identity to the *ppa *of *U. urealyticum, M. mycoides *ssp *mycoides*, and *M. capricolum *ssp *capricolum *were calculated to be 59.7%, 58.7%, and 58.3%, respectively. Figure 2 shows an alignment of sPPases of selected *Mycoplasma *species. The characteristic signature of sPPase which is essential for the binding of cations was identified at amino acid positions 54 to 60 (Figure 2) using the program PREDICT PROTEIN http://cubic.bioc.columbia.edu/predictprotein/. Possible signatures for sPPases are D[SGDN]D[PE][LIVMF]D[LIVMGAG]. The signature of the *M. suis *sPPase was determined as DGDPLDV (amino acids are underlined in the universal signature; Figure 2). The 13 conserved residues which build the active site of sPPases could be identified in the *M. suis *sPPase, too (Figure 2).

### Expression of recombinant PPase in *E. coli*

**Figure 3:**
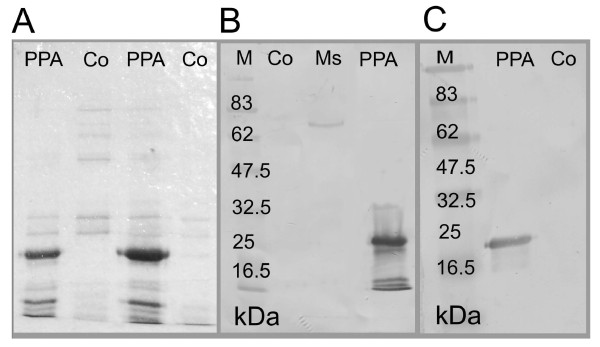
**Expression and immunological characterization of the *M. suis *sPPase**. (A) Coomassie-stained SDS polyacrylamide gel electrophoresis of recombinant *M. suis *PPase., Co, non-induced IMAC purified *E. coli *lysate; PPA, IMAC purified recombinant PPase. (B) Immunoblot analysis of recombinant PPase and *M. suis *whole cell antigen; immunological detection with anti-PPase rabbit immune serum; M, molecular weight standard; PPA, recombinant PPase; Ms, purified *M. suis *cells; Co, non-induced IMAC purified *E. coli *lysate. (C) Immunoblot of recombinant PPAse; immunological detection with a serum pool from experimentally infected pigs; PPA, recombinant PPase; Co, non-induced IMAC purified *E. coli *lysate.

The entire ORF of the *M. suis ppa *was assembled as a synthetic gene and one UGA_Trp _codon at position 274-276 was replaced by UGG. Other changes in the synthetic *ppa *were done to optimize the sequence for the heterologous *E. coli *expression. Induction of *E. coli *transformants containing the *ppa *gene resulted in the high-level expression of a 20 kDa-protein as shown in Figure 3A. Recombinant PPase was used to raise a PPase-specific rabbit polyclonal antiserum. The specificity of the rabbit serum was demonstrated by probing an immunoblot containing purified rPPase and a *M. suis *preparation. The anti-PPase serum reacted clearly with a single band of 20 kDa corresponding to the purified rPPase. In the *M. suis *preparations a weak band of 20 kDa and a clear band of 80 kDa potentially corresponding to a tetrameric form of the *M. suis *PPase were detected (Figure 3B). No reaction could be observed neither with the blood control preparation of *M. suis *negative pigs nor the non-induced *E. coli *control.

### Characterization of PPase in *M. suis*

In order to prove the conserved existence of the PPase gene in *M. suis*, 25 *M. suis *isolates (20 isolates from domestic pigs and five isolates from wild boars) were screened by PCR. All isolates revealed a PCR amplification product of the expected size of approximately 500 bp. Sequence analysis of ten *ppa *PCR products revealed 100% sequence identity with the determined *M. suis ppa *sequence (Accession number FN394679).

To determine the antigenicity of the PPase of *M. suis *we analyzed convalescent serum pools from experimentally infected pigs by immunoblotting. All convalescent serum pools reacted clearly with rPPase. No reaction could be observed with sera taken from *M. suis *negative pigs. A representative immunoblot is shown in Figure 3C.

### Functional characterization of recombinant *M. suis *PPase

**Figure 4:**
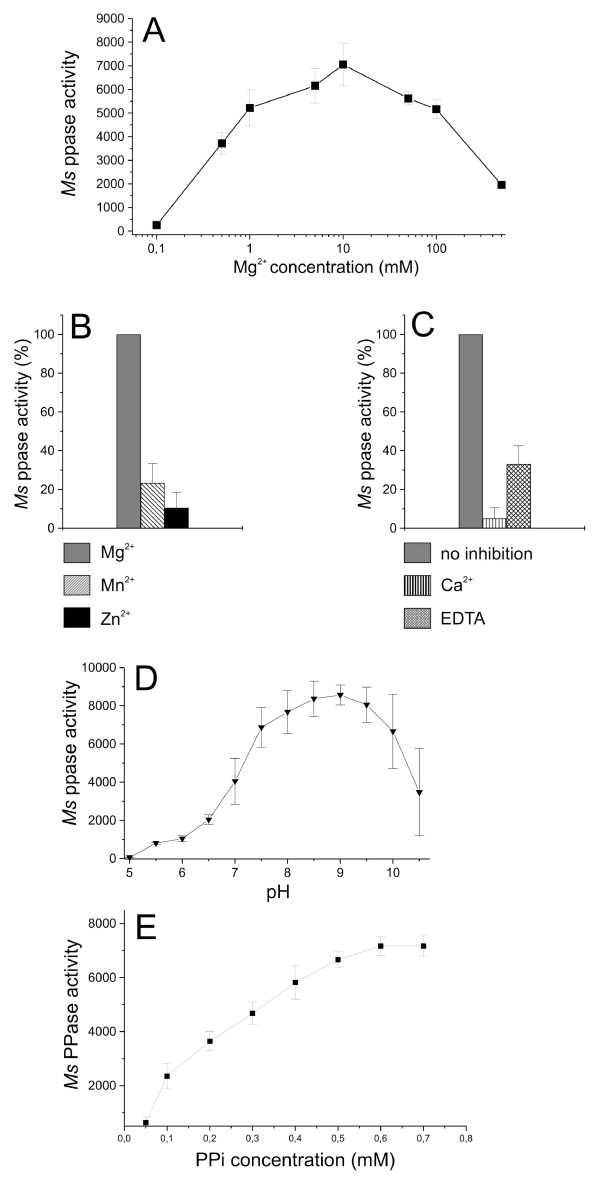
**Functional characterization of the recombinant *M. suis *sPPase**. (A) Activation of *M. suis *rPPase by Mg^2+^. The rPPase (10 ng/μl) was incubated for 5 min in the same buffer containing different concentrations of MgCl_2_. Values represent mean values ± standard deviation of five independent experiments. (B) Differences in the activation of rPPase by Mg^2+^, Mn^2+^, or Zn^2+^. Recombinant PPase (10 ng/μl) was incubated for 5 min in the same buffer containing 5 mM MgCl_2_, 5 mM MnCl_2 _and 5 mM MgCl_2_, respectively. Activation of *M. suis *rPPase by MgCl_2 _was set as 100%. Values represent mean values ± standard deviation of triplicates. (C) Inhibition of *M. suis *rPPase activity by Ca^2+ ^and EDTA. Recombinant PPase (10 ng/μl) was incubated for 5 min in buffer containing 5 mM MgCl_2 _alone and with 5 mM CaCl_2 _and 5 mM EDTA, respectively. Activity value of *M. suis *rPPase with MgCl_2 _alone was set as 100%. Values represent mean values ± standard deviation of triplicates. (D) pH value dependency of the *M. suis *rPPase activity. PPase activity was measured using 50 mM MgCl_2 _and buffers with increasing pH values. Data represent mean values ± standard deviation from five independent experiments. (E) Activity of *M. suis *rPPase using different PPi concentrations. Activity was measured with fixed concentrations of rPPase (10 ng/μl) and 50 mM MgCl_2 _at a pH of 9.0. Values represent mean values ± standard deviation of five independent experiments.

The dependency of the *M. suis *PPase activity on the pH value was determined between pH 5 and 10.5. As shown in Figure 4D the optimum pH for the *M. suis *PPase activity was observed at pH 9.0. At conditions below pH 7.5 and above pH 10.0 its activity decreased considerably.

The effect of different Mg^2+ ^concentrations on the *M. suis *PPase activity is shown in Figure 4A. High enzyme activity was found between 1 and 100 mM Mg^2+ ^with a maximum activity at a concentration of 10 mM Mg^2+^. Performing the reaction at a pH of 7.5 the maximum PPase activity was found at a concentration of 50 mM. Using an Mg^2+ ^depleted reaction buffer the *M. suis *PPase-mediated PPi hydrolysis was nearly abolished. Substitution of Mg^2+ ^cations with Mn^2+ ^and Zn^2+ ^resulted in significantly lower activities of 25.34% ± 12.1%, and 14.3% ± 9.5% respectively of the Mg^2+ ^induced activity (Figure 4B).

To further characterize the *M. suis *PPase the effect of inhibitors on the activity was evaluated. Enzymatic activity was inhibited more than 95%, and 70% in the presence of 5 mM Ca^2+ ^and 5 mM EDTA, respectively (Figure 4C).

## Discussion

In this study, we identified, for the first time, a gene encoding the sPPase of one representative of the uncultivable hemotrophic mycoplasma group, i.e. *M. suis*. PPase plays an important role in the bacterial energy metabolism [11,12] and is the enzyme responsible for the hydrolysis of pyrophosphate which is formed principally as the product of many biosynthetic reactions that utilize ATP. Since our knowledge on the metabolism of *M. suis *and other hemotrophic mycoplasmas is rather limited enzymes associated with their metabolism are of our special interest.

The *M. suis *ORF encoding the sPPase showed a typically low G+C content of 30.11% which lies within the normal range of other mycoplasmas [19,20]. The identified *M. suis *sPPase signature sequence which is responsible for the cation binding was identical to those of *M. mycoides *ssp *mycoides *and *M. capricolum *ssp *capricolum*. Furthermore, all functionally important active site residues could be identified in the *M. suis *sPPase. Interestingly, the *M. suis *sPPase is considerably shorter than other mycoplasma sPPases (164 *vs*. 180-185 amino acid residues) due to differences in the C-terminal region. State-of-the-art knowledge on the uncultivable hemotrophic mycoplasmas does not allow for a statement as to which function the absence of amino acid residues on the C-terminus might incur. There could be a possible relevance for its subcellular localization. Additionally, the *ms*262 clone harbors a second ORF encoding a putative *M. suis *thioredoxin. The thioredoxin system operates via redox-active disulphides and provides electrons for a wide range of metabolic processes in prokaryotic cells. Especially within the genus *Mycoplasma *the thioredoxin complex apparently belongs to the metabolic core reactions [21,22]. Comparison of the genome structures flanking the *ppa *ORF with the sequenced *Mycoplasma *species revealed no homologies (data not shown).

After heterologous expression of the sPPase in *E. coli *the protein was found in the cytoplasm with a molecular weight of 20 kDa. In *M. suis *whole cell preparations the sPPase was detected as a 20 kDa band to a minor degree. Predominantly the enzyme was found to have a molecular weight of approx. 80 kDa indicating that the *M. suis *sPPase obviously consists of four subunits. Since the inference that the *M. suis *sPPase is tetrameric is solely based on the results of an immunoblot using anti-rPPase antibodies (Fig 3B) the final proof of the tetrameric form has to be provided as soon as an *in vitro *cultivation of *M. suis *is possible. For other Mycoplasmas nothing is known about the protein properties of sPPase since they have only been identified via their DNA sequences. However, other studies report that most eubacterial PPases are homohexamers [23,24], and, as is unusual, sometimes homotetramers e.g. *Aquifex aeolicus *[25,26] or *Rhodospirillum rubrum *[27]. Where molecular phylogeny is concerned the *Mycoplasma *sPPases are clustered with the cyanobacteria within the prokaryotic Family I PPase lineage [27]. The *M. suis *sPPase showed characteristic properties in terms of cation requirement: Mg^2+ ^confers the highest efficiency in activating the *M. suis *sPPase in a concentration-dependent manner. Other cations (Zn^2+ ^and Mn^2+^) could replace Mg^2+^, but the effectiveness of the latter cations was significantly lower. Furthermore, Ca^2+ ^and EDTA inhibited the enzyme for catalysis. These results support the conclusion that the *M.suis *sPPase belongs to the Family I PPases. Family I PPase has shown strong metal cation-dependency, with Mg^2+ ^conferring the highest efficiency [14] and sensitivity to inhibition by Ca^2+ ^[28]. In contrast, Family II PPase prefers Mn^2+ ^over Mg^2+ ^[17]. The most notable characteristic of the *M. suis *recombinant sPPase was its pH activity profile with an optimum at pH 9.0 since (i) optimal pH of most bacterial sPPases ranged from pH 5.0 to 8.0 [25], and (ii) the physiological blood pH value of pigs is 7.4 ± 0.4. Therefore, it is ambiguous which role the unusual pH optimum could play with regard to the pathogenesis of *M. suis *induced diseases. Moreover, no statement is possible about optimal pH ranges for other mycoplasmal sPPases since this study is the first functional characterization of a sPPase of a *Mycoplasma *species. For *M. suis *it is known that experimental induced acute diseases lead to severe hypoglycemia and blood acidosis with a mean pH value of 7.13 [29]. All these changes were considered to result from the high glucose consumption of *M. suis *during maximum bacteremia [1]. However, nothing is known about the changes in blood parameters during natural *M. suis *infections and especially during the chronic course of persistent infections with nearly physiological glucose metabolism. It has been reported from other infections, e.g. *Streptococcus pneumoniae*-infections in rats that infections could lead to significantly increased blood pH values [30].

Notably, infected pigs showed antibodies against recombinant sPPase. This may result from the sPPase being an ectoenzyme which might be located on the external surface. Alternatively, anti-*Ms *PPAse antibodies could be an outcome of bacterial lysis in the animal host. The first possibility is rather unlikely since no signal peptide was found in any *Mycoplasma *PPase and all other Familiy I PPases are clearly soluble and not secreted [27]. Probably sPPase could be one of the eight *M. suis *specific antigens which we have described recently [9].

## Conclusion

By using a screening of genomic libraries of uncultivable bacteria *M. suis *we were able to identify so far unknown components of the energy metabolism. We identified and characterized the inorganic pyrophosphatase of *M. suis*. Knowing the functional characteristics of such an essential enzyme may help to establish an *in vitro *cultivation system for hemotrophic mycoplasmas. Furthermore, as an antigenic and conserved protein *M. suis *sPPase could in future be further analyzed as a diagnostic antigen.

## Methods

### Bacterial strains and isolates, plasmids, and experimental porcine sera

*M. suis *cells were obtained from experimentally infected pigs as previously described [31,32]. *E. coli *K12 strains were Top10 and LMG194 (Invitrogen, Basel, Switzerland). For DNA manipulation and protein expression the plasmids pUC19 (Roche-Diagnostics, Rotkreuz, Switzerland) and pBad*Myc*His (C-terminal His- and *Myc*-tag, Invitrogen) were used. Experimental sera and *M. suis *isolates were available from previous studies [33,34].

### DNA extraction, library construction and sequence analysis

DNA extraction of *M. suis *was performed as previously described [31]. Customized DNA library construction was performed by Medigenomix (Martinsried, Germany). *M. suis *DNA fragments averaging from 1.5 kb to 3.0 kb were ligated into the pUC19 vector. In order to detect *M. suis *sequences 300 clones were randomly selected for DNA-sequencing. Customized sequencing was performed by Medigenomix. Nucleotide sequences were analyzed by using the FASTA aligorithm (Biocomputing service, University Zurich, http://www.bio.unizh.ch. For determination of putative open reading frames we used an ORF finder program http://www.ncbi.nlm.nih.gov/projects/gorf/. Translation of ORFs to amino acid sequences was performed by taking into account the alternative genetic codon usage of mollicutes (UGA encodes tryptophan instead of stop).

### Hybridization analysis

Hybridization was performed as previously described [31]. Briefly, *M. suis *genomic DNA was digested with *Eco*RI, analyzed on a 0.8% agarose gel and transferred to Hybond-N nylon membranes by capillary transfer using 1.5 M NaCl, 0.25 M NaOH as transfer buffer. The *ppa*-containing library clone *ms*262 was digested with the restriction enzymes *Hind*III and *EcoR*I. Due to an internal *Eco*RI digestion site the insert was divided into two fragments of approx. 1200 bp and 800 bp. Both fragments were labeled with digoxigenin-dUTP (Roche-Diagnostics) and used as probes.

### Cloning, expression of *M. suis ppa *and purification of the recombinant enzyme

To account for the *Mycoplasma *specific use of the UGA codon as tryptophan the *ppa *sequence was adapted to the codon usage of *E. coli *and *de novo *synthesized (Medigenomix). The *de novo ppa *was ligated into the pBad*Myc*His vector (pBad-*ppa*) and transformed into *E. coli *LMG194.

Recombinant pBad-*ppa E. coli *clones were grown to an OD_600 nm _of 0.6 at 37°C. Protein expression was induced by the addition of 0.02% arabinose. *E. coli *cultures were further incubated for 2 h at 37°C. His-tagged proteins were purified by nickel affinity chromatography (Qiagen, Hombrechtikon, Switzerland) as previously described [9,10]. The purification of 2 liter culture yielded a total of 1 mg recombinant protein. Purity of protein was estimated as 90%. Non-induced cultures were prepared accordingly as controls for immunoblots and enzyme activity assays.

### Enzyme activity assay

Protein content was determined by the method of Bradford (BioRad, Reinach, Switzerland) using bovine serum albumin as a standard. The recombinant *M. suis *sPPase activity was assayed as described by Saheki and coworkers [35] using a reaction mixture containing 5 mM Mg^2+^, 100 mM Tris, pH 7.5 and 1 mM PP_i _(Na_4_P_2_O_7_) at 55°C in a final volume of 200 μl. Reactions were started by adding 10 μL diluted *M. suis *rPPase (100 ng) and stopped by adding 1 ml 200 mM Glycin/HCl, pH 3.0. Then, 125 μl of 1% ammonium molybdate (in 25 mM H_2_SO_4_) and 125 μl of 1% ascorbic acid (in 0.05% KHSO_4_) were added to the mixtures and incubated for 30 min at 37°C. Yeast sPPase (Sigma, Buchs, Switzerland) was used as positive control. Preparations derived from non-induced pBad-*ppa *(purified accordingly to recombinant PPase) were used as negative controls. To determine the Mg^2+ ^and pH dependency individual assay components were varied. Activity was also measured using 5 mM Mn^2+^, Zn^2+ ^instead of Mg^2+ ^cations. For inhibition assays 5 mM Ca^2+ ^and EDTA, respectively, were added to the reaction mixture. The amount of P_i _liberated from the hydrolysis of PP_i _was measured using a spectrophotometer (Shimadzu 160-UV-A) and a standard P_i _curve. The PPase activity was defined as μmol P_i _min^-1 ^mg^-1 ^protein.

### Preparation of an anti-PPase rabbit immune serum

A rabbit immune serum was prepared as previously described [10] using 0.4 mg recombinant PPase for each immunization. Immunizations were conducted under the registration number 156/2002 with the legal prescriptions.

### SDS PAGE and immunoblots

SDS PAGE and immunoblots were performed according to standard protocols. The *M. suis *cells were prepared from the blood of experimentally infected pigs as previously described [32]. Negative controls were accordingly prepared from the blood of *M. suis*-negative pigs.

### PCR and sequencing

PCR amplification of the *ppa *gene was performed using the primers: *ppa*_for: ATGTCAAAAAATAATATAGTGGA; *ppa*_rev TTAATAATTTGATTGTTATTCTCC, and the HotStarTaq Polymerase Master Mix (Qiagen). PCR conditions were: 15 min at 95°C for activation of Taq polymerase, 30 cycles of denaturation at 95°C for 30 s, annealing at 60°C for 30 s, and extension at 72°C for 1 min. Amplified fragments were purified using the Qiaquick PCR Purification Kit (Qiagen) and sequenced (Medigenomix).

The *ppa *sequence was deposited in the EMBL Nucleotide Sequence Database under accession number FN394679.

## Authors' contributions

KH-planned, developed and co-coordinated the project, analyzed the data, wrote the manuscript; SP-functional characterization; did the enzyme activity assays; MS-screened the *M. suis *genomic libraries, performed the hybridization experiments; MK-expressed the inorganic pyrophosphate in *E. coli*, performed SDS PAGE and immunoblots; MMW-contributed to the data analysis and manuscript preparation; KMF-performed enzyme activity assays, protein purification procedures, SDS PAGE and immunoblots; LEH-project design, manuscript preparation and project oversight.
